# Maternal metabolic adaptations are necessary for normal offspring growth and brain development

**DOI:** 10.14814/phy2.13643

**Published:** 2018-03-13

**Authors:** Angela M. Ramos‐Lobo, Isadora C. Furigo, Pryscila D. S. Teixeira, Thais T. Zampieri, Frederick Wasinski, Daniella C. Buonfiglio, Jose Donato

**Affiliations:** ^1^ Department of Physiology and Biophysics Institute of Biomedical Sciences University of São Paulo São Paulo SP Brazil

**Keywords:** Energy balance, glucose homeostasis, hypothalamus, metabolic programming

## Abstract

Several metabolic adaptations emerge during pregnancy and continue through lactation, including increases in food intake and body weight, as well as insulin and leptin resistance. These maternal adaptations are thought to play a role in offspring viability and success. Using a model of attenuated maternal metabolic adaptations induced by ablation of the *Socs3* gene in leptin receptor expressing cells (SOCS3 KO mice), our study aimed to investigate whether maternal metabolic changes are required for normal offspring development, and if their absence causes metabolic imbalances in adulthood. The litters were subjected to a cross‐fostering experimental design to distinguish the prenatal and postnatal effects caused by maternal metabolic adaptations. Males either born or raised by SOCS3 KO mice showed reduced body weight until 8 weeks of life. Both adult males and females born or raised by SOCS3 KO mice also had lower body adiposity. Despite that, no significant changes in energy expenditure, glucose tolerance or insulin resistance were observed. However, males either born or raised by SOCS3 KO mice showed reduced brain mass in adulthood. Furthermore, animals born from SOCS3 KO mice also had lower proopiomelanocortin fiber density in the paraventricular nucleus of the hypothalamus. In conclusion, these findings indicate that the commonly observed metabolic changes in pregnancy and lactation are necessary for normal offspring growth and brain development.

## Introduction

Pregnancy and lactation are exceptional moments, in which females not only need to attend their own energetic demands but also provide energy to their offspring (Ladyman et al. 2010; Woodside et al. 2012). Several metabolic adaptations emerge during these periods, helping to supply the additional energy required. For example, pronounced increases in food intake are observed during gestation and lactation (Ladyman et al. 2010; Woodside et al. 2012). Pregnancy also leads to insulin resistance which is thought to play a role to ensure a high placental glucose uptake (Catalano 2014). Furthermore, pregnant animals develop leptin resistance which is perceived by a reduced anorexigenic response to this hormone and by a lower leptin's capacity to recruit STAT3 signaling pathway in the hypothalamus in comparison to virgin animals (Ladyman and Grattan 2005, 2016; Ladyman et al. 2009, 2012; Trujillo et al. 2011; Nagaishi et al. 2014).

Leptin resistance has been better characterized in diet‐induced obese animals and this condition is a key feature of obesity (Balland and Cowley 2015). Some authors suggest that leptin resistance can emerge from defects in the transduction of leptin receptor (LepR) signaling pathways (Balland and Cowley 2015). Accordingly, obese animals show increased hypothalamic expression of regulatory proteins that inhibit LepR signaling (Balland and Cowley 2015). Among different factors, LepR signaling can be inhibited by the suppressor of cytokine signaling‐3 (SOCS3) (Bjorbaek et al. 1998). Obese animals show high SOCS3 expression in the hypothalamus and SOCS3 ablation in the brain or in specific neuronal populations prevents leptin resistance and partially protects against diet‐induced obesity (Mori et al. 2004; Kievit et al. 2006; Briancon et al. 2010; Reed et al. 2010; Matarazzo et al. 2012; Pedroso et al. 2014). Pregnant animals also exhibit increased hypothalamic SOCS3 expression (Zampieri et al. 2015). Notably, SOCS3 inactivation in LepR‐expressing cells prevents leptin resistance and attenuates the increases in food intake, adiposity, and insulin resistance typically observed in pregnant mice (Zampieri et al. 2015). Therefore, SOCS3 expression in LepR‐expressing cells coordinates maternal metabolic adaptations.

Although the metabolic adaptations of pregnancy and lactation are well described, the actual importance of these changes for the offspring's development is still unclear. A lower food intake during pregnancy and lactation could decrease the energy supply for the offspring, leading to reduced growth. Indeed, calorie or protein‐restricted diets during pregnancy or lactation impair offspring growth (Zhan et al. 2007; Teodoro et al. 2012; Aiken and Ozanne 2013). Previous studies have shown that either under or overnutrition during gestation and/or lactation can affect the predisposition of the offspring to metabolic diseases in adulthood, a concept known as metabolic programming (Ozanne 2001; Heerwagen et al. 2010). The formation of hypothalamic neurocircuits relevant to energy and glucose homeostasis is also sensitive to nutrient availability during pregnancy and lactation (Bouret et al. 2008; Vogt Merly et al. 2014). In this study, we studied mice deficient of SOCS3 in LepR‐expressing cells since these females have a diminished development of maternal metabolic changes (Zampieri et al. 2015, 2016). Using this mouse model, our study aimed to investigate the long‐term metabolic consequences in mice born and/or raised by females presenting attenuated pregnancy‐ and lactation‐induced metabolic adaptations. Thus, we tested whether maternal metabolic changes are required for normal offspring development and if their absence causes metabolic imbalances in adulthood.

## Material and Methods

### Animals

The deletion of *Socs3* gene in LepR‐expressing cells was achieved by breeding the LepR‐IRES‐Cre strain (B6.129‐Lepr^tm2(cre)Rck^/J, Jackson Laboratories) with the SOCS3‐floxed mouse (B6;129S4‐SOCS3^tm1Ayos^/J, Jackson Laboratories), as previously described and validated (Pedroso et al. 2014, 2016; Zampieri et al. 2015, 2016; Bohlen et al. 2016). Animals carrying the SOCS3 conditional deletion in LepR‐expressing cells (SOCS3 KO group) were homozygous for the loxP‐flanked *Socs3* and LepR‐Cre alleles. The control group was composed of littermate animals carrying only the LepR‐Cre allele in homozygosity. Control and SOCS3 KO mice were weaned at 3–4 weeks of age and their mutations were confirmed by genotyping the DNA that had been previously extracted from the tail tip (REDExtract‐N‐Amp™ Tissue PCR Kit, Sigma). Mice were maintained under standard conditions of light (12‐h light/dark cycle) and temperature (23 ± 1°C). All animal procedures were approved by the Ethics Committee on the Use of Animals of the Institute of Biomedical Sciences at the University of São Paulo, and were performed according to the ethical guidelines adopted by the Brazilian College of Animal Experimentation.

### Evaluation of gestation and lactation

In all experiments, virgin SOCS3 KO females were bred with control males, whereas control females were bred with SOCS3 KO males. Consequently, all pups carried a similar genotype (heterozygosity for the loxP‐flanked *Socs3* allele and homozygosity for the LepR‐Cre allele). After the detection of copulatory plugs, females were single‐housed and this was considered to be the first day of pregnancy. Food intake and body weight were monitored daily during the entire gestation and for 3 weeks during lactation. The litter size was recorded on the day of birth and standardized to five pups to ensure comparable metabolic demands during lactation. The offspring weight was determined on days 2, 8, and 12 of lactation. Postpartum maternal behaviors were assessed on days 8 and 12 of lactation. Before the behavioral test, the litter (5 pups) was weighted and separated from the mother for 4 h. Then, the litter was weighted again and distributed in the corners of the female's cage. We evaluated the time required to contact, retrieve, and group the pups into the nest and to crouch over them. After 1 h from the beginning of the test, the offspring were weighted, and the difference from the beginning to the end of the test represented the milk consumption. A subgroup of control and SOCS3 KO females were killed on the 12th day of lactation to collect the mammary tissue (inguinal gland) for gene expression analyses using a protocol described previously (Buonfiglio et al. 2015, 2016). The numbers of fetuses and uterine reabsorptions were analyzed in subgroups of control and SOCS3 KO females killed on the 16th day of gestation.

### Energy balance and glucose homeostasis of the offspring

To investigate the specific effects of either gestation or lactation, the litters were subjected to a cross‐fostering experimental design on the day of birth in order to produce four groups: (1) CON‐CON: mice born and raised by control females; (2) CON‐KO: mice born from control females, but raised by SOCS3 KO females; (3) KO‐CON: mice born from SOCS3 KO females, but raised by control females; and (4) KO‐KO: mice born and raised by SOCS3 KO females. We studied male and female mice consuming regular rodent chow (2.99 kcal/g; 9.4% calories from fat; Quimtia, Brazil), and a subgroup of male mice consuming high‐fat diet (HFD; 5.31 kcal/g; 58% kcal derived from fat; Pragsoluções, Brazil) from 8 weeks of age. Initially, the body weight was monitored weekly until approximately 30 weeks of age. Then, mice were single‐housed to evaluate food intake for 1 week. Next, mice were subjected to a glucose tolerance test (2 g glucose/kg; i.p.) and to an insulin tolerance test (1 IU insulin/kg; i.p.). Male mice on HFD were placed in the Oxymax/Comprehensive Lab Animal Monitoring System (CLAMS; Columbus Instruments, Columbus, OH, USA) to analyze O_2_ consumption, CO_2_ production, respiratory exchange ratio (RER), water intake, and locomotor activity (through infrared beam sensors). After 3 days of adaptation inside the CLAMS, the metabolic parameters of each mouse were evaluated for four consecutive days. Therefore, the results presented here were the average of this period. Mice were killed after 4 h of food deprivation to collect different fat depots (perigonadal, subcutaneous, and retroperitoneal) in order to determine body adiposity. The nose‐anus length and brain mass were also assessed in male mice consuming regular chow.

### Quantification of neuronal fibers

Subgroups of male mice consuming regular chow were perfused transcardially with saline, followed by 4% formaldehyde fixative solution. Subsequently, brains were cut in 30‐*μ*m‐thick sections using a freezing microtome. Brain sections of CON‐CON, CON‐KO, KO‐CON, and KO‐KO groups were subjected to immunofluorescence staining in order to evaluate the integrated optical density (IOD) of proopiomelanocortin (POMC) or agouti‐related peptide (AgRP) immunoreactive fibers in the paraventricular nucleus of the hypothalamus (PVH), dorsomedial nucleus of the hypothalamus (DMH), and lateral hypothalamic area (LHA). Briefly, brain sections were rinsed in 0.02 mol/L potassium PBS, pH 7.4 (KPBS), followed by incubation in 3% normal donkey serum for 1 h. Next, sections were incubated overnight in anti‐*β*‐endorphin antisera (1:2000; Phoenix Pharmaceuticals), since *β*‐endorphin is a POMC‐derived peptide, or anti‐AgRP antisera (1:2000, Phoenix Pharmaceuticals, Inc.). Subsequently, sections were incubated for 90 min in Alexa Fluor^488^‐conjugated secondary antibody (Jackson ImmunoResearch). After rinses in KPBS, sections were mounted onto gelatin‐coated slides and coverslipped using Fluoromount G mounting medium (E.M.S.). The IOD obtained in the PVH, DMH, and PVH were analyzed using the ImageJ software (http://rsb.info.nih.gov/ij) and subtracted by the IOD assessed in adjacent areas with low staining (background). One representative rostrocaudal level was analyzed for each hypothalamic nucleus. Brain sections were analyzed using a Zeiss Axioimager A1 microscope (Zeiss, Germany) and epifluorescence photomicrographs were captured using a Zeiss Axiocam HRc camera (Zeiss) and the Axiovision software (Zeiss; version 4.8.2). Image processing and lettering was carried out with the Photoshop software (Adobe Systems Inc., Mountain View, CA).

### Statistical analysis

The comparisons between control and SOCS3 KO females were performed using the unpaired two‐tailed Student's *t* test. The differences among CON‐CON, CON‐KO, KO‐CON, and KO‐KO groups were analyzed using one‐way or two‐way ANOVA, followed by Newman–Keuls post hoc test. GraphPad Prism software was used for the statistical analyses and the results were expressed as mean ± SEM. Only *P* values <0.05 were considered to be statistically significant.

## Results

### Maternal metabolic adaptations are attenuated in SOCS3 KO mice

**Figure 1:**
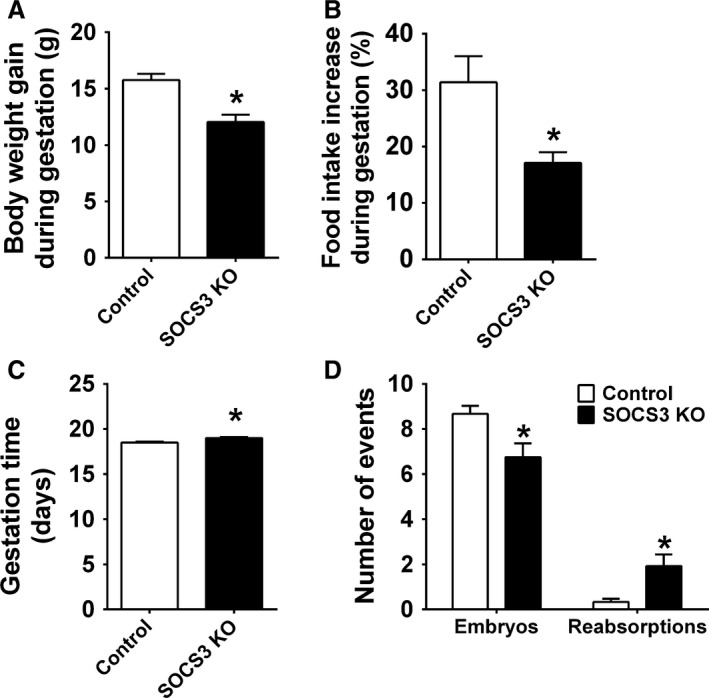
Ablation of SOCS3 in LepR cells leads to metabolic alterations in gestation. (A–B) Bar graphs comparing the increase in body weight (*n *=* *17–19/group) and food intake (*n *=* *9–17/group) during pregnancy in control and SOCS3 KO mice. (C) Bar graph comparing gestation time (days) in control (*n *=* *24) and SOCS3 KO mice (*n *=* *23). (D) Number of embryos and uterine reabsorptions of late pregnant control (*n *=* *12) and SOCS3 KO (*n *=* *12) mice. *Significantly different (*P *<* *0.05) compared to control group.

As previously described (Zampieri et al. 2015), ablation of SOCS3 in LepR‐expressing cells caused a significant decrease in weight gain and hyperphagia during pregnancy (Fig. 1A–B). Conditional SOCS3 deletion also increased pregnancy duration (Fig. 1C). The analysis of the uterus on the 16th day of gestation indicated a decreased number of fetuses and an equivalent increase in reabsorptions in mutant mice, compared to control animals (Fig. 1D). Despite the lower number of fetuses in pregnant SOCS3 KO mice, no significant differences in mean fetus weight (Control: 0.436 ± 0.036 g; SOCS3 KO: 0.391 ± 0.014; *P *=* *0.3034) or in total weight of fetuses (Control: 3.43 ± 0.48 g; SOCS3 KO: 2.30 ± 0.50; *P *=* *0.1383) were observed between the groups.

### Ablation of SOCS3 in LepR‐expressing cells decreased lactation performance

**Figure 2:**
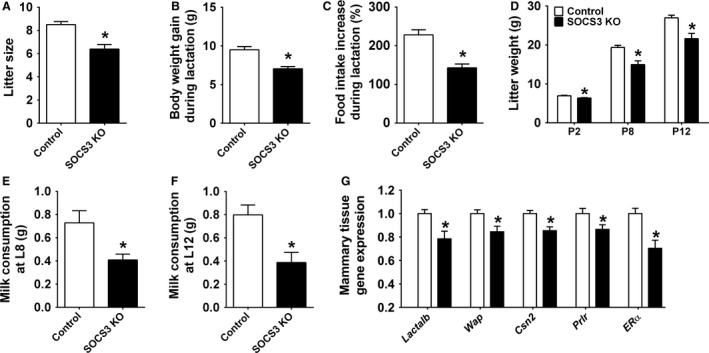
Ablation of SOCS3 in LepR cells causes metabolic alterations during lactation. (A) Litter size of control (*n *=* *30) and SOCS3 KO (*n *=* *28) mice. (B–C). Bar graphs comparing the increase in body weight (*n *=* *18–20/group) and food intake (*n *=* *6–8/group) during lactation in control and SOCS3 KO mice. (D) Body weight of litter of five pups at postnatal days 2, 8, and 12 from control (*n *=* *7) and SOCS3 KO dams (*n *=* *6). (E–F) Milk consumption of litter of five pups at postnatal days 8 (E) and 12 (F) of control (*n *=* *7) and SOCS3 KO mice (*n *=* *6). (G) Real‐time PCR to determine changes in mRNA expression in the mammary tissue (inguinal gland) of a subgroup of control (*n *=* *7) and SOCS3 KO (*n *=* *6) females that were killed on the 12th day of lactation. *Significantly different (*P *<* *0.05) compared to control group.

**Figure 3:**
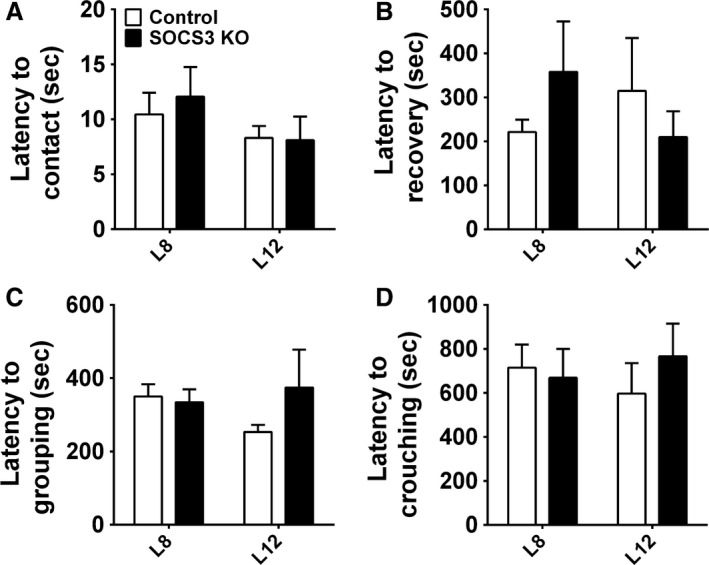
Postpartum maternal behavior in control and SOCS3 KO mice. (A–D) Latency to contact and retrieve all pups, to group them into the nest and to crouch over on days 8 and 12 of lactation (*n *=* *6–7/group).

In accordance with the lower number of fetuses during late pregnancy, SOCS3 KO mice had a smaller litter size on the day of birth (Fig. 2A). The sex ratio of the litters was not affected in SOCS3 KO mice since no significant difference between the percentage of male and female puppies was observed comparing SOCS3 KO mice and control mice (data not shown). Litter size was then standardized to five pups to ensure comparable metabolic demands during lactation. SOCS3 KO mice showed a reduced weight gain during lactation (compared to the body weight at the first day of pregnancy) and an attenuated increase in food intake throughout lactation (Fig. 2B–C). The reduced food intake during lactation probably affected the capacity of SOCS3 KO to meet the energetic demands of the pups, since the offspring weight gain was significantly reduced compared to control group (Fig. 2D). The impaired offspring growth in SOCS3 KO mice may be a consequence of a lower capacity to provide milk to the offspring, as shown on days 8 and 12 of lactation (Fig. 2E–F). An mRNA expression analysis was performed in the inguinal mammary tissue of mice on 12th day of lactation and we observed a decreased mRNA expression of *α*‐lactalbumin (Lactalb), whey protein (Wap), and *β*‐casein (Csn2), transcripts that encode major milk proteins, in SOCS3 KO mice compared to control animals (Fig. 2G). Additionally, the mRNA expression of the prolactin receptor (Prlr) and estrogen receptor *α* (ER*α*), key hormone receptors involved in mammopoiesis and lactogenesis, was also suppressed in the mammary gland of SOCS3 KO mice (Fig. 2G). Despite the decreased lactation performance exhibited by SOCS3 KO mice, maternal behavior was not affected by the conditional deletion (Fig. 3). Thus, control and SOCS3 KO mice showed similar latencies to contacting, recovering, grouping, and crouching the pups on days 8 and 12 of lactation (Fig. 3A–D).

### Changes in energy balance in mice born and/or raised by females presenting attenuated maternal metabolic adaptations

**Figure 4:**
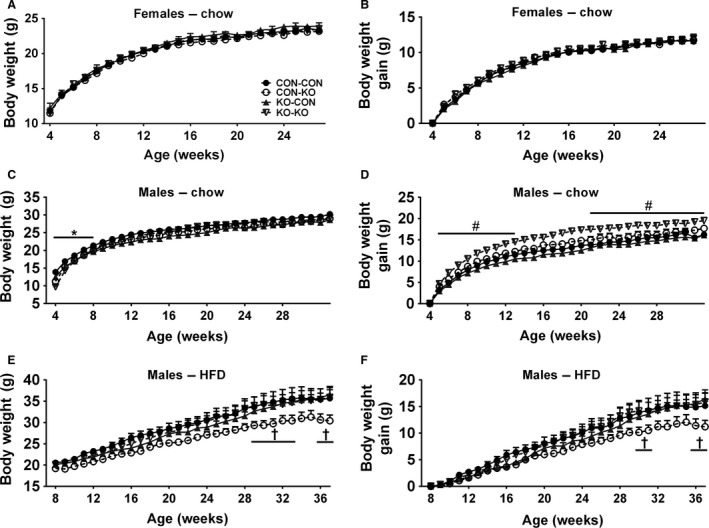
Body weight changes in females and males on regular chow, and in males on HFD. (A–B) Body weight (A) and body weight gain (B) of CON‐CON (*n *=* *26), CON‐KO (*n *=* *18), KO‐CON (*n *=* *11), and KO‐KO (*n *=* *21) females on regular chow from weaning until 30 weeks of age. (C–D) Body weight (C) and body weight gain (D) of CON‐CON (*n *=* *20), CON‐KO (*n *=* *7), KO‐CON (*n *=* *14), and KO‐KO (*n *=* *11) males on regular chow from weaning until 30 weeks of age. (E–F) Body weight (E) and body weight gain (F) of CON‐CON (*n *=* *10), CON‐KO (*n *=* *13), KO‐CON (*n *=* *13), and KO‐KO (*n *=* *11) males on HFD. Mice started to consume the HFD at the 8th week of life. *All groups are significantly different (*P *<* *0.05) compared to CON‐CON group. ^#^KO‐KO group is significantly different (*P *<* *0.05) compared to CON‐CON group. ^†^CON‐KO group is significantly different (*P *<* *0.05) compared to all group.

After characterizing the effects of SOCS3 ablation during pregnancy and lactation, we studied the long‐term metabolic consequences in the offspring. For this purpose, we initially investigated possible body weight changes in animals from CON‐CON, CON‐KO, KO‐CON, and KO‐KO groups. No significant differences in the body weight (Fig. 4A) and weight gain (Fig. 4B) among the groups of female mice were observed. In contrast, male mice either born or raised by SOCS3 KO mice (CON‐KO, KO‐CON, and KO‐KO groups) exhibited a lower body weight (*P *<* *0.05) until the 8th week of life compared to CON‐CON animals consuming regular chow (Fig. 4C). However, body weight of all groups became similar along time (Fig. 4C). When the weight gain was compared among the groups of male mice on regular chow, KO‐KO group showed the highest increase in comparison with the remaining groups (Fig. 4D). The diet‐induced obesity protocol started at the 8th week of life in subgroups of male mice (Fig. 4E–F). By this age the initial body weight difference among the groups was not statistically significant (Fig. 4E). Interestingly, the CON‐KO group exhibited an attenuated body weight and weight gain with time, compared to other groups consuming HFD (Fig. 4E–F).

**Figure 5:**
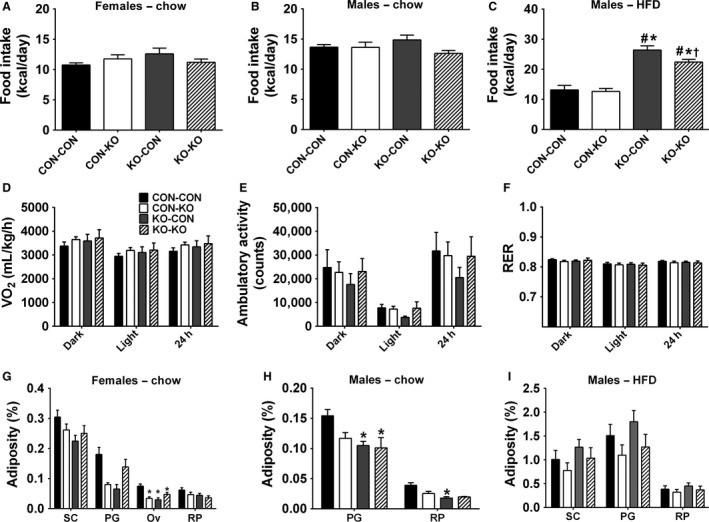
Food intake, energy expenditure, ambulatory activity, respiratory exchange ratio, and body adiposity. (A) Food intake in females on regular chow of CON‐CON (*n *=* *25), CON‐KO (*n *=* *17), KO‐CON (*n *=* *9), and KO‐KO (*n *=* *16) groups. (B) Food intake in males on regular chow of CON‐CON (*n *=* *10), CON‐KO (*n *=* *7), KO‐CON (*n *=* *12), and KO‐KO (*n *=* *11) groups. (C) Food intake in males on HFD of CON‐CON (*n *=* *8), CON‐KO (*n *=* *11), KO‐CON (*n *=* *12), and KO‐KO (*n *=* *11) groups. (D–F) Energy expenditure (VO_2_), ambulatory activity, and respiratory exchange ratio (RER) in males on HFD of CON‐CON, CON‐KO, KO‐CON, and KO‐KO groups (*n* = 5–7/group). These data represent the mean of five consecutive days and were presented as dark phase (from 8 pm to 8 am), light phase (from 8 am to 8 pm), and 24 h cycle. (G–I) Adiposity from subcutaneous (SC), perigonadal (PG), and retroperitoneal (RP) fat pads of (G) females on regular chow (CON‐CON *n *=* *21, CON‐KO *n *=* *5, KO‐CON *n *=* *9, and KO‐KO *n *=* *12), (H) males on regular chow (CON‐CON *n *=* *18 CON‐KO *n *=* *5, KO‐CON *n *=* *11, and KO‐KO *n *=* *4), and (I) males on HFD (CON‐CON *n *=* *8 CON‐KO *n *=* *12, KO‐CON *n *=* *12, and KO‐KO *n *=* *11).*Significantly different (*P *<* *0.05) compared to CON‐CON group. ^#^Significantly different (*P *<* *0.05) compared to CON‐KO group. ^†^Significantly different (*P *<* *0.05) compared to KO‐CON group.

After monitoring body weight, baseline food intake was assessed. No differences were observed among the experimental groups consuming regular chow (Fig. 5A–B). Among male mice on HFD, KO‐CON had significantly higher food intake compared to all groups. In addition, KO‐KO group also showed increased food intake compared to CON‐CON and CON‐KO groups (Fig. 5C). Energy expenditure, spontaneous ambulatory activity, and respiratory exchange ratio were also analyzed in male mice on HFD, but no significant differences were observed among the groups (Fig. 5D–F). The perigonadal or periovarian fat depots of CON‐KO, KO‐CON, and KO‐KO females were significantly lighter than those of CON‐CON female mice (Fig. 5G). KO‐CON and KO‐KO males consuming regular chow also exhibited a lower body fat accumulation compared to CON‐CON animals (Fig. 5H). However, in male mice consuming HFD, no significant differences in body adiposity were observed among the groups (Fig. 5I).

### Similar glucose tolerance and insulin sensitivity in mice born and/or raised by females presenting maternal metabolic adaptations

**Figure 6:**
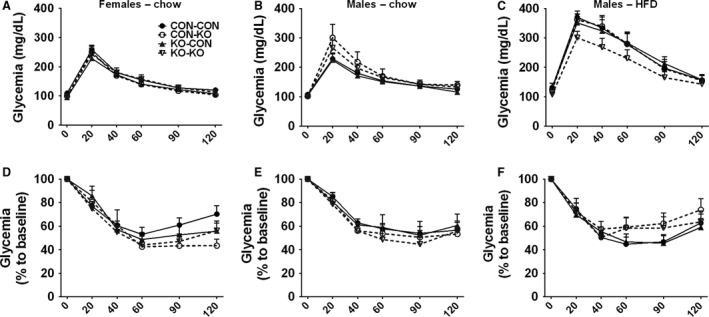
Glucose homeostasis of CON‐CON, CON‐KO, KO‐CON, and KO‐KO groups. (A–C) Glucose tolerance test of (A) females on regular chow (CON‐CON *n *=* *25 CON‐KO *n *=* *9, KO‐CON *n *=* *17, and KO‐KO *n *=* *16), (B) males on regular chow (CON‐CON *n *=* *10 CON‐KO *n *=* *12, KO‐CON *n *=* *7, and KO‐KO *n *=* *11), and (C) males on HFD (CON‐CON *n *=* *6 CON‐KO *n *=* *10, KO‐CON *n *=* *11, and KO‐KO *n *=* *9). (D–F) Insulin tolerance test of females on regular chow (D), males on regular chow (E), and males on HFD (F).

The glucose homeostasis was also evaluated in the experimental animals. However, no significant differences in glucose tolerance were observed among the groups of female or male mice consuming regular chow (Fig. 6A–B), or in male mice on HFD (Fig. 6C). Subsequently, an insulin tolerance test was performed. In accordance with previous results, no differences in the insulin sensitivity were observed among the experimental groups (Fig. 6D–F).

### Changes in brain mass and AgRP and POMC fibers

**Figure 7:**
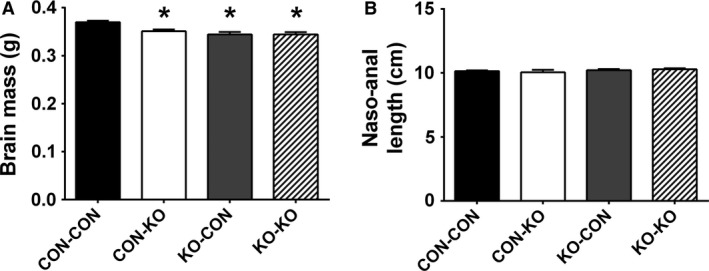
Brain mass and body length in males on regular chow. (A–B) Bar graphs comparing the brain mass (A) and the naso‐anal length (B) in CON‐CON (*n *=* *18), CON‐KO (*n *=* *5), KO‐CON (*n *=* *12), and KO‐KO (*n *=* *9) male mice on regular chow. *Significantly different (*P *<* *0.05) compared to CON‐CON group.

**Figure 8:**
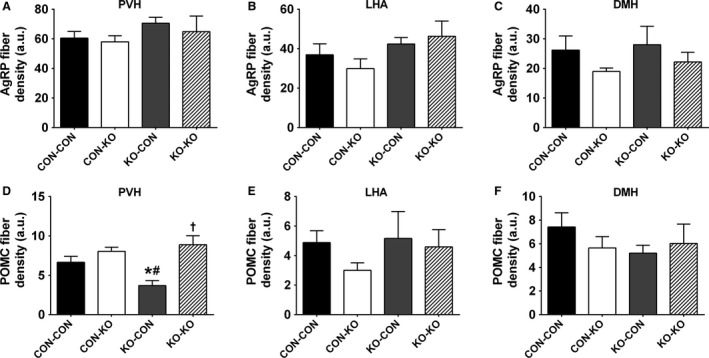
Axonal projections of ARH neurons. (A–C) Bar graph comparing AgRP fiber density in the PVH (A), LHA (B), and DMH (C) in males consuming regular chow (*n *=* *7/group). (D–F) Bar graph comparing POMC (*β*‐endorphin) fiber density in the PVH (D), LHA (E), and DMH (F) in males consuming regular chow (*n *=* *6/group). *Significantly different (*P *<* *0.05) compared to CON‐CON group. ^#^Significantly different (*P *<* *0.05) compared to CON‐KO group. ^†^Significantly different (*P *<* *0.05) compared to KO‐CON group.

**Figure 9:**
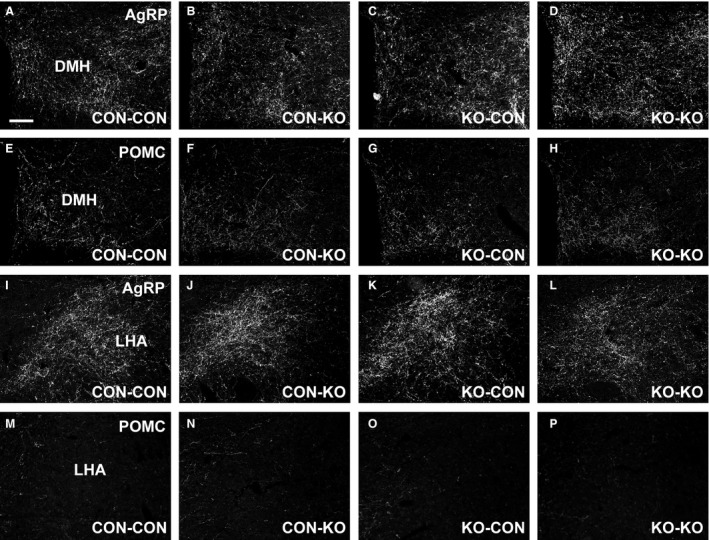
AgRP and POMC fibers in the DMH and LHA. (A–D) Representative photomicrographs of AgRP fibers in the DMH of CON‐CON (A), CON‐KO (B), KO‐CON (C), and KO‐KO (D) males on regular chow. (E–H). Representative photomicrographs of POMC (*β*‐endorphin) fibers in the DMH of CON‐CON (E), CON‐KO (F), KO‐CON (G), and KO‐KO (H) males on regular chow. (I–L) Representative photomicrographs of AgRP fibers in the LHA of CON‐CON (I), CON‐KO (J), KO‐CON (K), and KO‐KO (L) males on regular chow. (M–P) Representative photomicrographs of POMC fibers in the LHA of CON‐CON (M), CON‐KO (N), KO‐CON (O), and KO‐KO (P) males on regular chow. Scale bar = 100 *μ*m.

**Figure 10:**
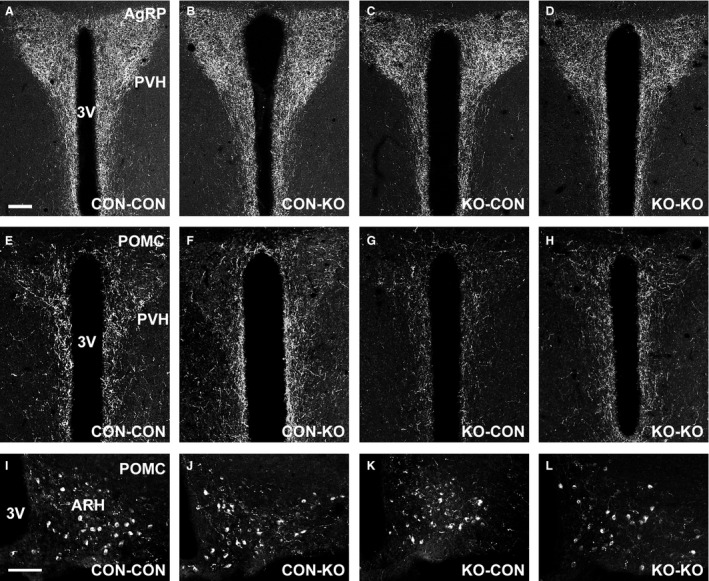
POMC but not AgRP fibers in the PVH are altered by maternal SOCS3 ablation in LepR cells. (A–D) Representative photomicrographs of AgRP fibers in the PVH of CON‐CON (A), CON‐KO (B), KO‐CON (C), and KO‐KO (D) males on regular chow. (E–H) Representative photomicrographs of POMC (*β*‐endorphin) fibers in the PVH of CON‐CON (E), CON‐KO (F), KO‐CON (G), and KO‐KO (H) males on regular chow. (I–L) Representative photomicrographs of POMC neurons in the ARH of CON‐CON (I), CON‐KO (J), KO‐CON (K), and KO‐KO (L) males on regular chow. 3V, third ventricle. Scale bars = 100 *μ*m.

Nutrient availability during intrauterine or perinatal periods not only can induce long‐term metabolic alterations, but it may also cause permanent changes in brain development (Bouret et al. 2008; Vogt Merly et al. 2014). Therefore, we evaluated the brain mass in male mice consuming regular chow. Notably, CON‐KO, KO‐CON, and KO‐KO males showed a significant reduction in brain mass compared to CON‐CON animals (Fig. 7A). This difference was observed despite similar body weight (Fig. 4C) and naso‐anal length (Fig. 7B) at the time of tissue collection. Next, the density of AgRP and POMC fibers was evaluated in several hypothalamic nuclei of male mice consuming regular chow (Figs. 8, 9, and 10). The density of AgRP fibers to PVH, LHA, and DMH were not different among the groups (Figs. 8A–C, 9A–D, I–L, and 10A–D). Although no changes in the density of POMC fibers were observed in the LHA and DMH (Figs. 8E–F and 9E–H, M–P), a reduction in POMC fibers in the PVH was found in the KO‐CON group (Figs. 8D and 10E–H). This reduction was not caused by changes in the number of POMC neurons in the arcuate nucleus of the hypothalamus (ARH; CON‐CON: 28.7 ± 1.6 cells; CON‐KO: 31.4 ± 2.5 cells; KO‐CON: 25.3 ± 1.4 cells; KO‐KO: 27.4 ± 2.3 cells; *P *=* *0.2199; Fig. 10I–L).

## Discussion

Since gestation and lactation are situations of high‐energy demand (Augustine et al. 2008; Woodside et al. 2012), several metabolic adaptations emerge during these periods, supposedly to improve offspring viability and success. However, the actual importance of these metabolic adaptations for normal offspring development has not been fully evaluated. Furthermore, it is also unclear whether the absence of maternal metabolic changes can predispose the offspring to metabolic imbalances in adulthood. Therefore, we developed a mouse model in which females exhibit attenuated maternal metabolic adaptations via inactivation of the *Socs3* gene in LepR‐expressing cells (Zampieri et al. 2015, 2016). These females showed reduced food intake and weight gain during pregnancy and lactation. As previously described (Zampieri et al. 2015), these females also have improved leptin and insulin sensitivity, leading to lower body fat accumulation during these periods. In this manuscript, we observed that attenuation of maternal metabolic changes caused a significant impact on the offspring. SOCS3 KO females showed a reduced number of embryos and smaller litter size. This reduction was not likely caused by decreased ovulation, since a concomitant increase in the number of uterine reabsorptions was found in late pregnant SOCS3 KO mice. Thus, in a uterine environment with less available energy, a reduction in the number of viable embryos seems to be a positive adaptation to ensure the successful birth of fewer pups. However, although some studies have shown reduced litter size in protein‐ or energy‐restricted dams (Young and Widdowson 1975), others found no significant influence on the number of pups on the day of birth (Kang‐Lee and Harper 1975; Park et al. 1994). Thus, a lower number of viable embryos found in SOCS3 KO females may not be caused only by decreased energy availability during gestation. Since previous studies have shown that SOCS3 regulates inflammatory responses in the placenta and embryonic lethality (Roberts et al. 2001; Robb et al. 2005), possible changes in placental SOCS3 expression in our conditional knockout model could have led to a reduced number of viable embryos.

In this study, we used homozygous animals for the LepR‐IRES‐Cre allele in both control and conditional knockout group. Our intention was to improve Cre‐mediated recombination since LepR transcription is normally very low and reports in the literature indicates an enhanced LepR‐IRES‐Cre‐mediated recombination in homozygous animals, reflecting more accurately the expected pattern of LepR expression in the brain (Leinninger et al. 2009; Leshan et al. 2009). Although high levels of Cre activity may result in nonspecific recombination (Morrison and Münzberg 2012) or cause brain development defects (Forni et al. 2006), these problems are unlikely in homozygous LepR‐IRES‐Cre mouse since no brain development defects have been observed in earlier studies (Pedroso et al. 2014, 2016; Zampieri et al. 2015, 2016; Bohlen et al. 2016). Additionally, in a previous study, we co‐localized a Cre‐mediated reporter protein (tdTomato) with leptin‐induced pSTAT3 using the LepR‐IRES‐Cre allele in homozygosity and we found a high degree of colocalization, which indicates that nonspecific recombination does not occur significantly in homozygous LepR‐IRES‐Cre (Nagaishi et al. 2014). Finally, a breeding strategy was chosen to guarantee a similar phenotype among the pups which carried the loxP‐flanked *Socs3* allele in heterozygosity and the LepR‐IRES‐Cre allele in homozygosity. Whether this genotype could affect the observed results remains unknown.

Lactation performance was impaired in SOCS3 KO females, leading to reduced offspring weight gain. Since no defects in postpartum maternal behavior were observed in SOCS3 KO females, the reduced offspring growth is likely explained by a lower capacity to produce milk. Accordingly, the mammary gland of SOCS3 KO dams showed lower expression of key proteins involved in mammopoiesis and lactogenesis, and their offspring consumed less milk during the 1‐hour test period. A massive caloric intake, along with the use of body fat reserves, is required to meet the high‐energy demands of a mouse offspring (Woodside et al. 2012). Thus, reduction in food intake during lactation became maladaptive in SOCS3 KO mice, possibly restricting the energy supply for the offspring. Thus, our findings suggest that SOCS3 ablation in LepR cells affected neuronal populations involved in energy balance control (Ramos‐Lobo and Donato 2017), but had no effect in neural circuits that regulate maternal behavior. This result is in accordance with the lack of LepR‐expressing cells and consequently SOCS3 ablation in key brain areas associated with parental care (Scott et al. 2009; Dulac et al. 2014; Nagaishi et al. 2014).

By evaluating the metabolic consequences in mice born and/or raised by SOCS3 KO mice, we observed that males were more affected than females. Evidence in the literature in fact indicates a sexually dimorphic impact in the metabolism and immune system caused by dietary changes during pregnancy and lactation (Zambrano et al. 2006; Oertelt‐Prigione 2012; Makarova et al. 2013; Sanchez‐Garrido et al. 2013; Sardinha et al. 2013; Morselli et al. 2016). Of note, higher morbidity and mortality rate during early life have been reported in males than in females (Hammond 1965; Naeye et al. 1971; Wells 2000), and this sexually dimorphic bias depends on caloric availability (Williams and Gloster 1992). Thus, since males are more vulnerable to early life stressors than females (Wells 2000), males may also be more prone to metabolic programming and other developmental deficiencies. Accordingly, while neonatal overfeeding influences the development of hypothalamic connectivity in male rats (Sominsky et al. 2017), female rats subjected to a similar nutritional manipulation showed no significant changes (Ziko et al. 2017). Shortly after weaning (4 weeks old), males either born or raised by SOCS3 KO animals exhibited lower body weight compared to mice born and raised by control dams. This result supports the idea that limited energy supplies either prenatally or postnatally impaired offspring growth in male mice born or raised by SOCS3 KO mice. Therefore, our mouse model caused a type of undernutrition, especially in male pups, which is not dependent of food restriction, dietary manipulations and other stressors, but induced by a reduction in maternal metabolic adaptations. Therefore, while the natural increases in food intake, body adiposity, and insulin resistance observed during pregnancy or lactation could be considered metabolic stressors to the female's organism, these adaptations produce intrauterine and postnatal benefits for the offspring. Consequently, our findings confirm the general assumption that the typical metabolic changes in pregnancy and lactation are indeed required for normal offspring development.

Previous studies that investigated dietary‐induced metabolic programming found long‐term consequences in glucose homeostasis (Chen et al. 2009; Vogt Merly et al. 2014). However, no differences in glucose tolerance or insulin sensitivity were observed in CON‐KO, KO‐CON, and KO‐KO groups, compared to CON‐CON animals. On the other hand, energy balance was affected by our genetic manipulation. Although male mice from CON‐KO, KO‐CON, and KO‐KO groups exhibited lower body weight until 8 weeks of life, these animals were able to recover the body weight, indicating a catch‐up, particularly in the KO‐KO group consuming regular chow. Nevertheless, CON‐KO, KO‐CON, and KO‐KO groups still showed lower body adiposity compared to CON‐CON animals. In males on HFD, the only notable differences were an increased food intake in KO‐CON and KO‐KO groups, as well as a reduced body weight in the CON‐KO group, although no statistically differences among the groups were observed in body adiposity, energy expenditure, ambulatory activity or RER. The lack of additional metabolic changes, despite the differences in weight gain, body adiposity or food intake in some groups, may be caused by the time these measurements were performed (after the long‐term body weight analysis). Thus, it is possible that younger animals could exhibit other metabolic alterations, but these changes became harder to detect in older mice. In addition, several variables that also affect the energy balance were not evaluated in this study. For example, changes in meal pattern (Nicklas et al. 2001; Farley et al. 2003) or microbiota (Turnbaugh et al. 2006; Shen et al. 2013) can have a significant impact in the metabolism, without necessarily affecting food intake or energy expenditure. Thus, future studies are still necessary to investigate in more detail the primary cause of the differences in weight gain, body adiposity or food intake observed in some experimental groups. Overall, despite the clear indication of reduced growth and undernutrition in pups born or raised by SOCS3 KO dams, no evidence of metabolic programming was found. Therefore, the attenuation of maternal metabolic adaptations seems to be insufficient to cause metabolic disorders in the offspring later in life.

We found a remarkable reduction in the brain mass of CON‐KO, KO‐CON, and KO‐KO males compared to CON‐CON animals. Therefore, either intrauterine or postnatal deficiencies can affect brain development in mice born or raised by SOCS3 KO mice, although no additive or synergic effect was found because KO‐KO group showed a similar brain mass compared to CON‐KO or KO‐CON animals. Dietary protein restriction is able to reduce brain growth in mice (Hoppe et al. 2007). Leptin‐deficient mice also have a reduced brain mass, which can be reverted by early leptin administration (Bereiter and Jeanrenaud 1979; Ahima et al. 1999). Since CON‐KO, KO‐CON, and KO‐KO males showed reduced body weight until 8 weeks of life and remained leaner even in adulthood, the reduced maternal metabolic adaptations possibly led to hypoleptinemia, which may have affected brain development. The long‐term consequences of the reduced brain mass in these animals are uncertain, but it is possible that several neurological functions may have been compromised. For example, maternal dietary changes can disturb hippocampal gene expression, learning, and memory function in the offspring (Page et al. 2014). In addition, future studies could investigate what regions account for the difference in brain mass among the experimental groups.

Former studies have also indicated that the formation of hypothalamic neurocircuits involved in energy balance is sensitive to nutrient availability during pregnancy and lactation (Bouret et al. 2008; Vogt Merly et al. 2014; Johnson et al. 2016). Thus, in this study the density of AgRP and POMC fibers to post synaptic targets was also investigated since this innervation represents important neurocircuits that regulate body weight and other metabolic aspects (Ramos‐Lobo and Donato 2017), and they are particularly affected by nutritional cues during development (Bouret et al. 2004, 2008; Vogt Merly et al. 2014; Johnson et al. 2016). We found a reduced density of POMC fibers in the PVH of KO‐CON males. This defect was not caused by changes in the number of POMC cells in the ARH. Interestingly, the KO‐CON groups consuming regular chow also exhibited the lowest body adiposity compared to the other groups, indicating that the absence of metabolic changes during pregnancy was critical for these deficiencies. Therefore, the reduced density of POMC fibers in the PVH of KO‐CON mice probably reflects their undernutrition and development deficiencies, rather than being the cause of their lower body adiposity.

## Conclusions

In summary, our findings provide robust evidence that the commonly observed metabolic changes during pregnancy and lactation are necessary for normal offspring development. The absence of maternal metabolic adaptations caused long‐term consequences in the energy balance and brain development of the offspring. However, mice born or raised by SOCS3 KO females presented a phenotype closer to that of undernutrition, rather than exhibiting evidence of metabolic programming that could lead to metabolic disorders in adulthood. Altogether, these findings contribute to the understanding of the importance of an adequate nutrition during pre‐ and postnatal periods, and how situations of altered maternal metabolic adaptations could have an impact in offspring development and metabolism.

## Conflict of Interest

The authors declare no conflicts of interest.
